# Synthesis and Gas Permeability of Polynorbornene Dicarboximides Bearing Sulfonyl Moieties

**DOI:** 10.3390/polym18010062

**Published:** 2025-12-25

**Authors:** Alejandro Onchi, Lisandra Rubio-Rangel, Arlette A. Santiago, Brian Omar Marín-Méndez, Mar López-González, Joel Vargas

**Affiliations:** 1 Instituto de Investigaciones en Materiales, Unidad Morelia, Universidad Nacional Autónoma de México, Antigua Carretera a Pátzcuaro No. 8701, Col. Ex Hacienda de San José de la Huerta, Morelia C.P. 58190, Michoacán, Mexico; aonchi@pceim.unam.mx (A.O.); rubiorangell@pceim.unam.mx (L.R.-R.); 2 Escuela Nacional de Estudios Superiores, Unidad Morelia, Universidad Nacional Autónoma de México, Antigua Carretera a Pátzcuaro No. 8701, Col. Ex Hacienda de San José de la Huerta, Morelia C.P. 58190, Michoacán, Mexico; arlette_santiago@enesmorelia.unam.mx; 3 Facultad de Ingeniería Química, Universidad Michoacana de San Nicolás de Hidalgo, General Francisco J. Múgica s/n, Morelia C.P. 58060, Michoacán, Mexico; 1341678h@umich.mx; 4 Instituto de Ciencia y Tecnología de Polímeros (ICTP-CSIC), Juan de la Cierva, 3, 28006 Madrid, Spain

**Keywords:** ROMP, polynorbornene dicarboximide, gas permeability, membrane

## Abstract

This work reports on the synthesis and ring-opening metathesis polymerization (ROMP) of two novel homologous sulfonyl-containing norbornene dicarboximide monomers, specifically, *N*-4-(trifluoromethylsulfonyl)phenyl-norbornene-5,6-dicarboximide (**1a**) and *N*-4-(trifluoromethylsulfonyl)phenyl-7-oxanorbornene-5,6-dicarboximide (**1b**) using the Grubbs 2nd generation catalyst (**I**). The polymers are thoroughly characterized by nuclear magnetic resonance (NMR), Fourier transform infrared spectroscopy (FT-IR), thermomechanical analysis (TMA), thermogravimetric analysis (TGA), atomic force microscopy (AFM), and X-ray diffraction (XRD), among other techniques. A comparative study of gas transport in membranes based on these ROMP-prepared polymers is performed and the gases studied are hydrogen, oxygen, nitrogen, carbon dioxide, methane, ethylene and propylene. It is found that the presence of sulfonyl pendant groups in the polymer backbone increases the gas permselectivity in slight detriment of the gas permeability compared to a polynorbornene dicarboximide lacking sulfonyl groups. The membrane of the sulfonyl-containing polymer with an oxygen heteroatom in the cyclopentane ring, **2b**, is also found to have one of the largest permselectivity coefficients reported to date for the separation of H_2_/C_3_H_6_ in glassy polynorbornene dicarboximides.

## 1. Introduction

Gas separation is a research topic of the utmost importance due to its relevance in industrial applications in several sectors, such as oil and gas, chemistry, petrochemicals, medicine, the environment and the food and beverage industry, among others [1,2]. Some gas separation technologies include cryogenic distillation, liquid or chemical absorption, pressure swing adsorption or vacuum adsorption (PSA, VSA), and adsorption on solid materials. However, some of the above require high energy consumption, maintenance, and complex equipment [3,4,5]. Membrane technology for gas separation is a process in which the gas dissolves in the membrane and diffuses through it at a certain rate, driven by the pressure difference on each side of the membrane [6]. It is considered an exceptional method for gas separation due to its simple operating mechanism, high efficiency, low costs and energy demands, lower environmental impact, and the possibility of chemically adapting the membranes to specific applications [7,8]. It is worth noting that gas separation membranes are mainly manufactured from polymeric materials.

In this sense, polynorbornene derivatives are attractive materials for gas separation membranes due to their remarkable thermo-mechanical and gas transport properties; in addition, they can be obtained by ring-opening metathesis polymerization (ROMP) as well as addition polymerization, resulting in different polymeric materials (polyalkenamers and polyolefins) with, in turn, different gas transport properties [9,10,11]. In particular, norbornene dicarboximide monomers can be easily modified, thereby facilitating the synthesis of homologous series of functionalized polymers, both linear [12,13] and cross-linked [14,15]. Likewise, polynorbornene dicarboximides also exhibit easy modification of their polymer backbones, including post-polymerization reactions, which facilitates obtaining membranes with tailored properties for various purposes [16,17,18].

The introduction of heteroatoms into the polymer main chain is another tool for tailoring physicochemical properties such as hydrophilicity [19,20,21], metal adsorption [22,23,24] and the uptake of other elements such as iodine [25,26,27], increasing electron density [28], tunning the energy gap in conducting polymers [29,30] and, of course, improving gas transport properties, among others [31]. For instance, the introduction of oxygen containing groups to the polynorbornene structure, such as epoxy, has improved selectivity over voluminous gases such as CO_2_ due to the molecular interactions between carbon dioxide molecules and the present ether bonds [32,33].

The sulfonyl group is the basis of polysulfones, a class of amorphous thermoplastic polymers characterized by their high thermal resistance and superior mechanical strength. This class of polymers has been shown to form membranes that are attractive for gas separation applications [34,35]. Another approach to enhance gas separation performance in polymer membranes involves mixing two polymers with different physicochemical properties and several studies have addressed this issue with a mixed polymer composed of polysulfone and polyimide [36,37]. The outcomes indicate that these mixed polymer membranes can provide higher gas permeability and improved membrane features compared to pure polymers in simple membranes [38,39,40]. Since imide moieties tend to compact polymer chains, the addition of bulky sulfonyl groups decreases intermolecular chain interactions and semicrystalline structure, thereby increasing free volume and improving gas permeability [41,42,43].

This study aims to elucidate the fundamental structure–property relationships governing gas permeability in a novel homologous series of functionalized polynorbornene dicarboximides synthesized via ROMP and comprehensively characterized by Fourier transform infrared spectroscopy, nuclear magnetic resonance, thermomechanical analysis, thermogravimetric analysis, X-ray diffraction, atomic force microscopy, and related techniques. In particular, we examine how the deliberate incorporation of sulfonyl pendant groups and an oxygen heteroatom within the cyclopentane ring modulates the physicochemical properties of the macromolecular architecture. Although polynorbornene-based structures have been addressed in gas separation, the synergistic effect arising from the combination of an electron-withdrawing sulfonyl group with an oxygen-bridging heteroatom in polynorbornene dicarboximides has remained largely unexplored in the membrane literature. This strategy enables the decoupling and assessment of the influence of oxygen heteroatom-induced chain packing efficiency and the strong electron-withdrawing nature of the sulfonyl moiety on membrane gas permeability. To the best of our knowledge, this work represents the first study employing these specific sulfonyl-containing polynorbornene dicarboximides for gas separation membranes, thereby establishing a new framework for the rational design of membranes with enhanced gas permselectivity.

## 2. Experimental Part

### 2.1. Characterization Techniques

The FT-IR spectra were recorded using a Thermo Scientific Nicolet iS10 FT-IR spectrometer (Thermo Fisher Scientific, Waltham, MA, USA) equipped with an ATR accessory and a diamond crystal, over the range of 4000–650 cm^−1^ at a spectral resolution of 4 cm^−1^. The ^1^H-NMR, ^13^C-NMR and ^19^F-NMR spectra were recorded on a Bruker Avance III HD spectrometer (Bruker AXS GmbH, Karlsruhe, Germany) at 400, 100 and 370 MHz, respectively, using CDCl_3_ and DMF-*d*_7_ as solvents. Tetramethylsilane (TMS) and hexafluorobenzene were employed as internal standards for the NMR analysis. The glass transition temperature (*T_g_*) was measured with a TA Instruments Thermomechanical Analyzer TMA Q400 (TA Instruments, New Castle, DE, USA) using a 10 °C/min heating rate under nitrogen atmosphere between 30 °C and 400 °C. The decomposition temperature (*T_d_*) was measured with a TA Instruments Thermogravimetric analyzer TGA 5500 (TA Instruments, New Castle, DE, USA) using a 10 °C/min heating rate under nitrogen atmosphere between 30 °C and 800 °C. A universal mechanical testing machine (Instron 1125-5500R, Instron, Norwood, MA, USA) was used to measure the Young’s modulus (*E*) and tensile strength (*σ*), employing a 50 kg load cell and a crosshead speed of 10 mm/min. The density of the polymers was determined by the flotation method, in ethanol at ambient conditions, with a Sartorius analytical balance model Quintix 124-1s (Sartorius AG, Göttingen, Germany). The X-ray diffractograms were obtained in a Bruker diffractogram model D2-Phaser 2nd Generation (Bruker AXS GmbH, Karlsruhe, Germany) using CuK_α_ radiation from 7 to 70° in the 2θ scale, at 30 Kv and 10 mA. The atomic force microscopy images of the polymer films were obtained in a Veeco Multimode SPM AFM (Veeco Instruments, Plainview, NY, USA) using the tapping mode.

### 2.2. Reagents

Norbornene-5,6-dicarboxylic anhydride (NDA) was prepared via the Diels–Alder cycloaddition reaction between maleic anhydride and cyclopentadiene, following literature procedures [14]. 1,2-Dichloroethane was dried and distilled over CaH_2_. Acetic anhydride, dichloromethane, chloroform, hexanes, methanol, ethanol, *N*,*N*-dimethylformamide (DMF) and anhydrous sodium acetate were used without further purification. 4-(Trifluoromethylsulfonyl)aniline, furan, tricyclohexylphosphine [1,3-bis(2,4,6-trimethylphenyl)-4,5-dihydroimidazol-2-ylidene][benzylidene] ruthenium dichloride (**I**), as well as other reagents, were acquired from Merck Sigma-Aldrich (St. Louis, MO, USA) and used as received.

### 2.3. Synthesis of Monomers

#### 2.3.1. Synthesis of Monomer *N*-4-(Trifluoromethylsulfonyl)phenyl-norbornene-5,6-dicarboximide (**1a**)

**Scheme 1:**
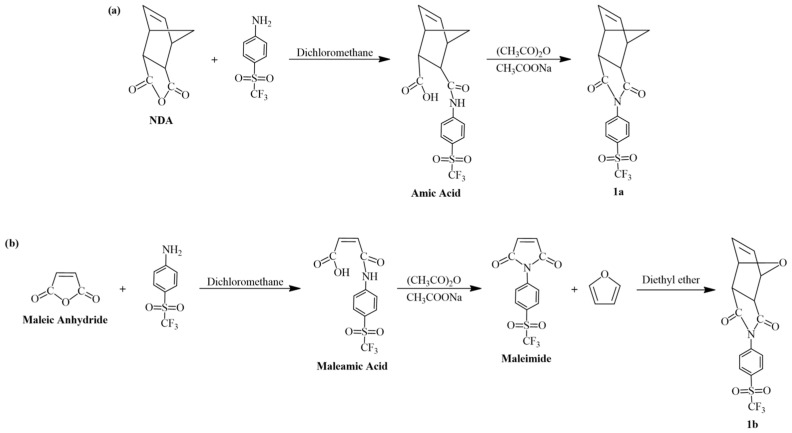
Synthesis of the sulfonyl-containing monomers: (**a**) **1a** and (**b**) **1b**, respectively.

0.2 g (0.88 mmol) of 4-(trifluoromethylsulfonyl)aniline and 0.1457 g (0.88 mmol) of NDA were dissolved in 20 mL of dichloromethane. The solution was maintained under reflux conditions for 24 h and an amic acid was obtained. The product was separated by filtration and dried at room temperature. Then, it was dissolved in 20 mL of acetic anhydride along with 2.0 g of anhydrous sodium acetate. This solution was maintained for 24 h at 65 °C, then allowed to cool to room temperature. The resulting product was precipitated in ice water and washed several times. The obtained monomer **1a** was recovered by filtration and recrystallized twice in hexanes (Scheme 1a). Yield: 36%. Melting point: 153–155 °C.

FT-IR (powder, cm^−1^): ν 3100 (C=C–H), 2990 (C–H, asym.), 2891 (C–H, sym.), 1773 (C=O, asym), 1701 (C=O, sym.), 1586 (C=C), 1376 (C–N), 1301 (S=O), 1169 (C–F), 698 (C–S).

^1^H-NMR (400 MHz, CDCl_3_, ppm): δ 8.06–7.55 (4H, m), 7.64–7.52 (*endo*, m), 6.37 (2H, s), 6.26 (*endo*, s), 3.59–3.32 (2H, m), 3.56–3.47 (*endo*, m), 2.98–2.87 (2H, m), 1.70–1.58 (1H, m), 1.81 (*endo*, d), 1.49–1.36 (1H, m).

^13^C-NMR (100 MHz, CDCl_3_, ppm): δ 175.99 (C=O), 139.18 (C–S), 138.08 (C=C), 132.19 (C–H, ar.), 130.38 (C–N), 127.89 (C–H, ar.), 124.55 (C–F), 121.31 (C–F), 118.07 (C–F), 114.83 (C–F), 47.96 (C–H), 46.09 (C–H), 43.10 (CH_2_).

^19^F-NMR (370 MHz, CDCl_3_, ppm): δ −78.09.

#### 2.3.2. Synthesis of Monomer *N*-4-(Trifluoromethylsulfonyl)phenyl-7-oxanorbornene-5,6-dicarboximide (**1b**)

0.2 g (0.88 mmol) of 4-(trifluoromethylsulfonyl)aniline and 0.0862 g (0.88 mmol) of maleic anhydride were dissolved in 20 mL of dichloromethane. The solution was maintained under reflux conditions for 24 h and a maleamic acid was obtained. The product was separated by filtration and dried at room temperature. Then, it was dissolved in 20 mL of acetic anhydride along with 2.0 g of anhydrous sodium acetate. This solution was maintained for 24 h at 65 °C, then allowed to cool to room temperature. The resulting maleimide was precipitated in ice water and washed several times. The maleimide was recovered by filtration and recrystallized twice in hexanes. Next, the maleimide and furan were dissolved in diethyl ether at a molar ratio of 1:1. The solution was maintained under stirring at room temperature for 5 days, and the precipitation of a white powder was observed. The obtained monomer **1b** was collected by filtration and recrystallized twice in ethanol (Scheme 1b). Yield: 31%. Melting point: 137–139 °C.

FT-IR (powder, cm^−1^): ν 3108 (C=C–H), 3026 (C–H, asym.), 2987 (C–H, sym.), 1777 (C=O, asym), 1712 (C=O, sym.), 1592 (C=C), 1362 (C–N), 1309 (S=O), 1172 (C–F), 1085 (C–O), 693 (C–S).

^1^H-NMR (400 MHz, CDCl_3_, ppm): δ 7.88–7.62 (4H, m), 6.44 (2H, s), 5.26 (2H, s), 2.91 (2H, s).

^13^C-NMR (100 MHz, CDCl_3_, ppm): δ 174.37 (C=O), 139.00 (C–S), 136.83 (C=C), 131.66 (C–H, ar.), 130.69 (C–N), 127.31 (C–H, ar.), 124.89 (C–F), 121.33 (C–F), 118.09 (C–F), 115.26 (C–F), 81.67 (C–H), 47.70 (C–H).

^19^F-NMR (370 MHz, CDCl_3_, ppm): δ −78.09.

### 2.4. Polymerization of the Sulfonyl-Containing Monomers

ROMP of the sulfonyl-containing monomers was conducted in 25 mL round bottom flasks under a dry nitrogen atmosphere. Ethyl vinyl ether was used in small amounts to inhibit polymerization. White polymer fibers formed upon pouring the polymer solution into excess acidified methanol under constant stirring at room temperature. The products were recovered by filtration and purified by solubilization in DMF and subsequent precipitation in stirring methanol. The polymers were finally dried under vacuum at 50 °C overnight.

#### 2.4.1. Synthesis of Poly(*N*-4-(Trifluoromethylsulfonyl)phenyl-norbornene-5,6-dicarboximide) (**2a**)

**Scheme 2:**
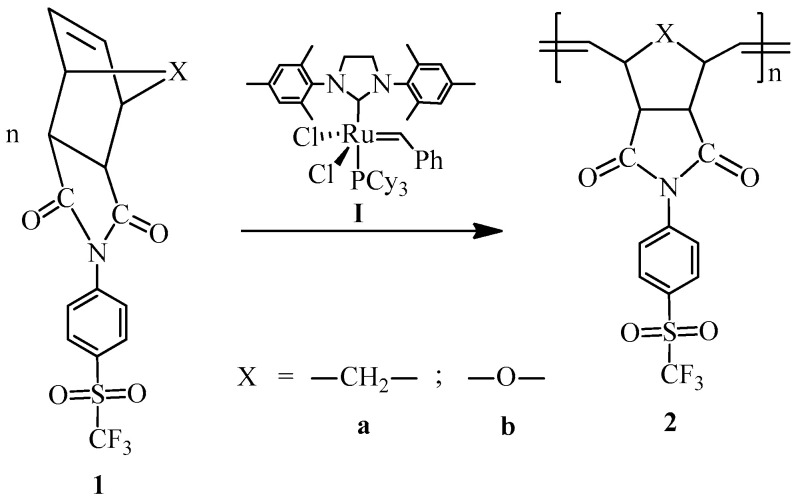
Synthesis of the sulfonyl-containing polymers via ROMP.

Monomer **1a** (0.2 g, 0.53 mmol) and catalyst **I** (0.449 × 10^−3^ g, 0.00053 mmol) were dissolved in 0.7 mL of 1,2-dichloroethane and stirred at 45 °C for 2 h. The resulting poly(*N*-4-(trifluoromethylsulfonyl)phenyl-norbornene-5,6-dicarboximide) (**2a**) was obtained as white fibers and is soluble in DMF (Scheme 2).

FT-IR (thin film, cm^−1^): ν 3101 (C=C–H), 2990 (C–H, asym.), 2896 (C–H, sym.), 1773 (C=O, asym), 1704 (C=O, sym.), 1597 (C=C), 1371 (C–N), 1302 (S=O), 1169 (C–F), 693 (C–S).

^1^H-NMR (400 MHz, DMF-*d*_7_, ppm): δ 8.69–7.97 (4H, m), 6.04 (1H, s, *trans*), 5.84 (1H, s,*cis*), 4.05–3.43 (2H, m), 3.42–3.02 (2H, m), 2.36 (1H, s), 1.88 (1H, s).

^13^C-NMR (100 MHz, DMF-*d*_7_, ppm): δ 176.92 (C=O), 141.26 (C–S), 134.44 (C=C), 131.72 (C–H, ar.), 129.41 (C–N), 128.89 (C–H, ar.), 124.94 (C–F), 121.70 (C–F), 118.46 (C–F), 115.23 (C–F), 51.70 (C–H), 45.85 (C–H), 43.30 (CH_2_).

^19^F-NMR (370 MHz, DMF-*d*_7_, ppm): δ −81.70.

#### 2.4.2. Synthesis of Poly(*N*-4-(Trifluoromethylsulfonyl)phenyl-7-oxanorbornene-5,6-dicarboximide) (**2b**)

Monomer **1b** (0.2 g, 0.53 mmol) and catalyst **I** (0.449 × 10^−3^ g, 0.00053 mmol) were dissolved in 0.7 mL of 1,2-dichloroethane and stirred at 45 °C for 2 h. The resulting poly(*N*-4-(trifluoromethylsulfonyl)phenyl-7-oxanorbornene-5,6-dicarboximide) (**2b**) was obtained as white fibers and is soluble in DMF (Scheme 2).

FT-IR (thin film, cm^−1^): ν 3106 (C=C–H), 3055 (C–H, asym.), 2970 (C–H, sym.), 1788 (C=O, asym), 1716 (C=O, sym.), 1592 (C=C), 1362 (C–N), 1301 (S=O), 1162 (C–F), 662 (C–S).

^1^H-NMR (400 MHz, DMF-*d*_7_, ppm): δ 8.49–7.88 (4H, m), 6.20 (1H, s, *trans*), 5.91 (1H, m, *cis*), 5.49–4.74 (2H, m), 3.78 (2H, s).

^13^C-NMR (100 MHz, DMF-*d*_7_, ppm): δ 174.60 (C=O), 140.64 (C–S), 131.74 (C–H, ar.), 132.16 (C=C), 129.31 (C–N), 128.83 (C–H, ar.), 124.77 (C–F), 121.51 (C–F), 118.28 (C–F), 115.03 (C–F), 77.28 (C–H), 53.20 (C–H).

^19^F-NMR (370 MHz, DMF-*d*_7_, ppm): δ −79.41.

### 2.5. Preparation of Membranes and Gas Permeability Experiments

Membranes were prepared by casting polymer solutions in DMF (~8 wt%) at 45 °C. After filtration, the solution was poured onto a glass plate and the solvent was allowed to evaporate slowly in a controlled DMF atmosphere. Next, the resulting polymer membranes were vacuum-dried at 100 °C overnight.

Gas permeation experiments were performed on a previously described system [14]. Gas permeation experiments were performed in a cell composed of two half-cells divided by the polymer membrane. A vacuum is created on both sides of the half-cells, followed by the addition of a gas at a fixed pressure to the high-pressure half-cell, also called the upstream half-cell. The upstream half-cell is connected to a Gometric pressure transducer. The flow of gas through the membrane into the low-pressure half-cell, also called the downstream half-cell, is monitored using an MKS 628/B pressure transducer. The entire permeation cell was placed in a thermostated water bath to ensure a stable temperature.

Given the downstream volume (*V*) in cm^3^, the area (*A*) of the membrane inside the half-cell in cm^2^, the membrane thickness (*l*) in cm, and the system pressures in cm Hg, the permeability coefficient (*P*) may be calculated according to the following expression:

(1) $$P=3.59\;\frac{Vl}{p_{0}AT}\underset{t\to o}{\lim}\left(\frac{dp}{dt}\right)$$

where *T* denotes the temperature in K, and *p* and *p*_0_ correspond to the downstream and upstream gas pressures, respectively. Permeability coefficients are expressed in barrer, with 1 barrer defined as 10^−10^ cm^3^ (STP) cm/(cm^2^s cmHg). The diffusion coefficient of the gas (*D*) is calculated using the following expression [44]:

(2) $$D=\;\frac{l^{2}}{6\theta }$$

where *θ* is the time lag, obtained from the *p* versus *t* isotherm plot. The units of the diffusion coefficient are cm^2^ s^−1^. The apparent solubility coefficient (*S*) can be expressed as follows:

(3) $$S=\;\frac{P}{D}$$

The units of the solubility coefficient are cm^3^(STP)/(cm^3^ cmHg).

## 3. Results and Discussion

**Figure 1:**
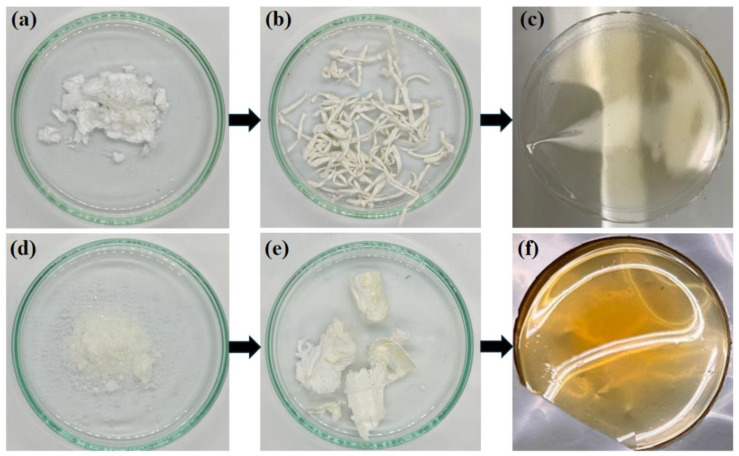
Photographic images of (**a**) monomer **1a**, (**b**) raw polymer **2a**, (**c**) polymer film **2a**, (**d**) monomer **1b**, (**e**) raw polymer **2b**, (**f**) polymer film **2b**.

On the one hand, monomer **1a** was successfully synthetized by reacting norbornene-5,6-dicarboxylic anhydride (NDA) with 4-(trifluoromethylsulfonyl)aniline to yield an amic acid that was then converted into an imide using acetic anhydride as a dehydrating agent. On the other hand, monomer **1b** was successfully synthetized by reacting maleic anhydride with 4-(trifluoromethylsulfonyl)aniline to obtain a maleamic acid, which was subsequently transformed into maleimide using the dehydrating agent mentioned above. The maleimide was then subjected to a Diels-Alder reaction with furan to obtain the norbornene dicarboximide monomer. The melting points of monomers **1a** and **1b** were found to be in the ranges of 153–155 °C and 137–139 °C, respectively. Next, the monomers were polymerized via ring-opening metathesis polymerization (ROMP) using the Grubbs 2nd generation Catalyst (**I**). The monomers reacted for 2 h, affording polymers in high yields (>98%). The experimental number average molecular weights (*M_n_*) and the molecular weight distributions (*MWD*) could not be determined due to the insolubility of the polymers in tetrahydrofuran (THF). However, the high yields obtained suggest the formation of high molecular weight polymers, and the theoretical molecular weight calculated from the monomer/catalyst molar ratio yields values of the order of 10^5^ g/mol. Figure 1 shows photographs of the sulfonyl-containing norbornene dicarboximide monomers prepared in this work, along with their corresponding raw polymers obtained via ROMP and the resulting films. It can be observed that the monomers are white crystals that gave rise to white fibrous polymers capable of forming resistant transparent films with a slightly yellowish coloration. The color change in the films could be related to the effect of charge transfer (CT) interaction formed by π electrons of ring arrangements in polynorbornene dicarboximides. One of these arrangements is preferred layer packing, in which neighboring imide rings are positioned adjacent to one another. Another arrangement is mixed-layer packing, which resembles a sandwich-like arrangement formed between imide groups and adjacent aromatic rings. DMF is a high-boiling-point solvent (153 °C). Therefore, its slow evaporation during membrane casting at 45 °C enhances polymer segment mobility and promotes stronger CT interactions, leading to changes in the molecular ordering arrangement of polynorbornene dicarboximide membranes. This effect has also been observed for linear polyimides [45].

**Figure 2:**
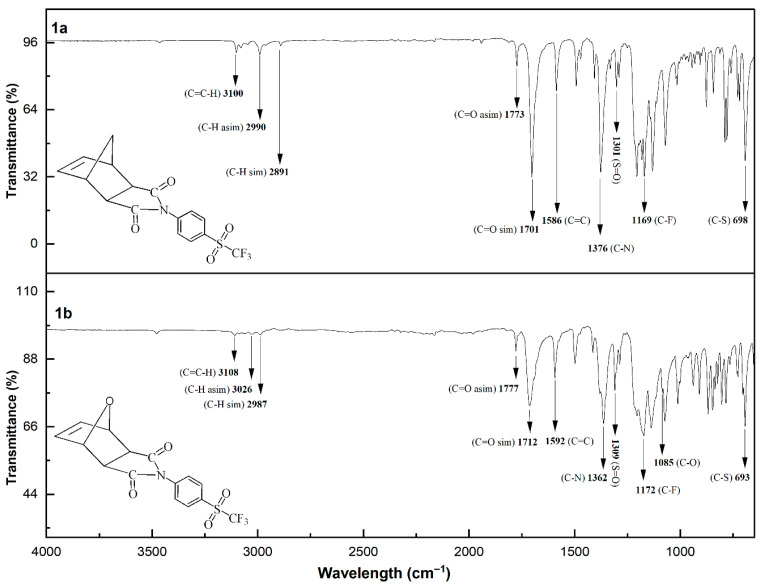
*FT-IR* spectra of the sulfonyl-containing norbornene dicarboximide monomers.

The chemical structures of the sulfonyl-containing norbornene dicarboximide monomers were confirmed by FT-IR spectroscopy, and the corresponding spectra are shown in Figure 2. The spectra are very similar to each other. In general, the following characteristic absorption bands can be observed: the aromatic H–C stretching vibration appears at approximately 3100 cm^−1^; the antisymmetric and symmetric stretching vibrations of the methylene C–H bonds are observed at 2990 and 2891 cm^−1^, respectively; the antisymmetric and symmetric stretching vibrations of the C=O bonds are located at 1773 and 1701 cm^−1^, respectively; the C=C stretching vibration is observed at 1586 cm^−1^; the C–N stretching vibration appears at around 1376 cm^−1^; the S=O stretching vibration is detected at approximately 1301 cm^−1^; the C–F stretching vibration is found near 1169 cm^−1^; the C–O stretching vibration appears at 1085 cm^−1^; and the band observed at approximately 698 cm^−1^ is attributed to the C–S stretching vibration.

**Figure 3:**
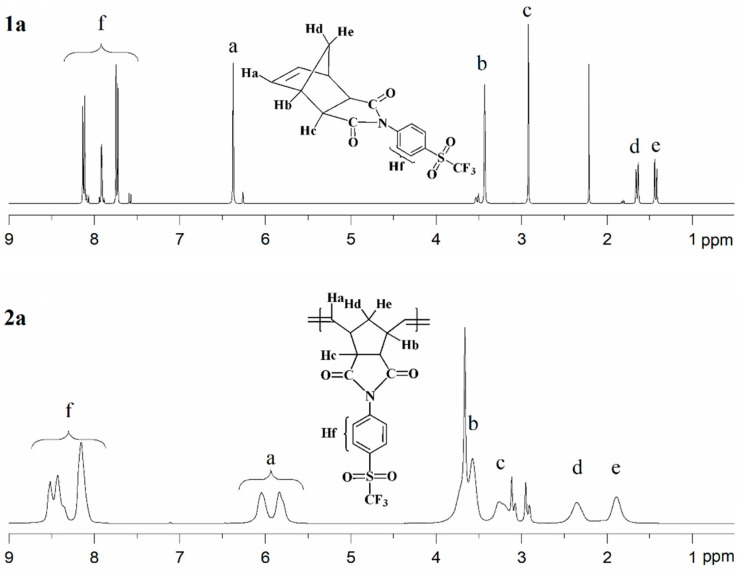
^1^H-*NMR* spectra of monomer **1a** (**top**) and polymer **2a** (**bottom**).

**Figure 4:**
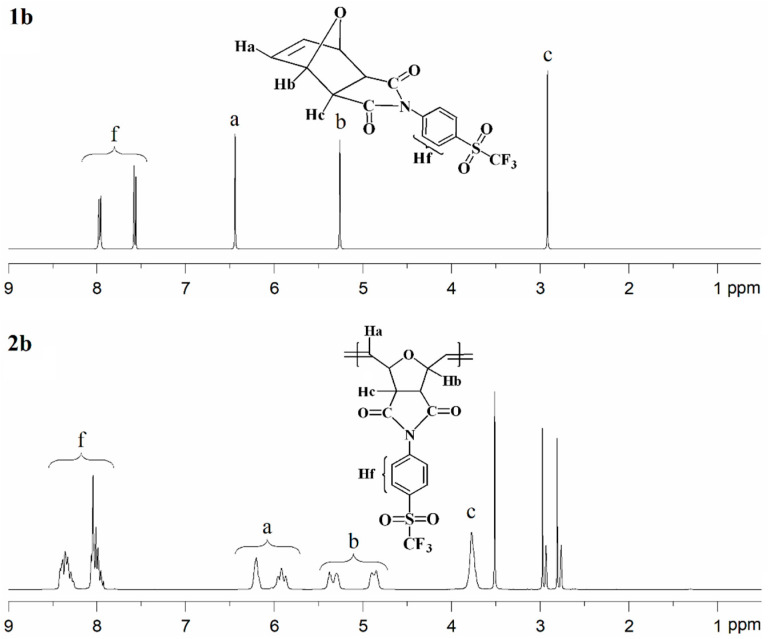
^1^H-*NMR* spectra of monomer **1b** (**top**) and polymer **2b** (**bottom**).

The chemical structures of the monomers and their corresponding polymers were confirmed by ^1^H, ^13^C, and ^19^F-NMR spectroscopy. The ^1^H-NMR spectra of monomer **1a** and its corresponding polymer **2a** are presented in Figure 3, while those of monomer **1b** and polymer **2b** are shown in Figure 4. The ^1^H-NMR spectrum of monomer **1a** (Figure 3, top) exhibits aromatic proton signals (H_f_) in the range of 8.06–7.55 ppm. The signal attributed to the olefinic protons (H_a_) appears at 6.37 ppm, whereas the resonances assigned to the –CH– protons (H_b_ and H_c_) are observed in the range of 3.59–2.87 ppm. The signals corresponding to the methylene protons (H_d_ and H_e_) are detected between 1.70 and 1.36 ppm. The ^13^C-NMR spectrum of monomer **1a** exhibits characteristic resonances for the carbonyl (C=O) carbons at approximately 175.9 ppm. Signals corresponding to the C–S carbons are observed at 139.1 ppm, while those attributed to the C=C carbons appear at 138.0 ppm. The aromatic C–H carbons give rise to signals at 132.1 and 127.8 ppm, and the C–N carbons are detected at 130.3 ppm. In addition, the –CF_3_ carbons display resonances at 124.5, 121.3, 118.0, and 114.8 ppm. The ^13^C-NMR spectrum of monomer **1a** also shows well-resolved aliphatic carbon signals at 47.9 and 46.0 ppm (C–H) and at 43.1 ppm (CH_2_). The ^19^F-NMR analysis revealed that the fluorine atoms in the –CF_3_ group of monomer **1a** are magnetically equivalent; accordingly, a single resonance was observed at approximately −78.09 ppm. The ^1^H-NMR spectrum of polymer **2a** is shown in Figure 3 (bottom). The signals assigned to the aromatic protons (H_f_) appear in the range of 8.69–7.97 ppm. The olefinic proton signal of the monomer at δ = 6.37 ppm is replaced by two new olefinic proton signals (H_a_) at δ = 6.04 ppm and δ = 5.84 ppm, corresponding to the *trans* and *cis* double bonds of the polymer main chain, respectively. The resonances attributed to the –CH– protons (H_b_ and H_c_) are observed in the range of 4.05–3.02 ppm, while the signals corresponding to the methylene protons (H_d_ and H_e_) are detected between 2.36 and 1.88 ppm.

The ^1^H-NMR spectrum of monomer **1b** is shown in Figure 4 (top). The aromatic proton signals (H_f_) are observed in the range of 7.88–7.62 ppm. The signal corresponding to the olefinic protons (H_a_) appears at 6.44 ppm, while the resonances assigned to the –CH– protons (H_b_ and H_c_) are detected in the range of 5.26–2.91 ppm. The ^13^C-NMR spectrum of monomer **1b** exhibits characteristic resonances for the carbonyl (C=O) carbons at approximately 174.3 ppm. Signals associated with the C–S carbons are observed at 139.0 ppm, while those attributed to the C=C carbons appear at 136.8 ppm. The aromatic C–H carbons give rise to signals at 131.6 and 127.3 ppm, and the C–N carbons are detected at 130.6 ppm. In addition, the –CF_3_ carbons show resonances at 124.8, 121.3, 118.0, and 115.2 ppm. The ^13^C-NMR spectrum of monomer **1b** also displays well-resolved aliphatic carbon signals at 81.6 and 47.7 ppm, corresponding to C–H carbons. The ^19^F-NMR analysis indicated that the fluorine atoms in the –CF_3_ group of monomer **1b** are magnetically equivalent; therefore, a single resonance was observed at approximately −78.09 ppm. The ^1^H-NMR spectrum of polymer **2b** is shown in Figure 4 (bottom). The signals attributed to the aromatic protons (H_f_) appear in the range of 8.49–7.88 ppm. The olefinic proton signal of the monomer at δ = 6.44 ppm is replaced by two new olefinic proton signals (H_a_) at δ = 6.20 ppm and δ = 5.91 ppm, corresponding to the *trans* and *cis* double bonds of the polymer main chain, respectively. The resonances assigned to the –CH– protons (H_b_) are observed in the range of 5.49–4.74 ppm, while the signal corresponding to the –CH– proton (H_c_) is detected at 3.78 ppm.

**Figure 5:**
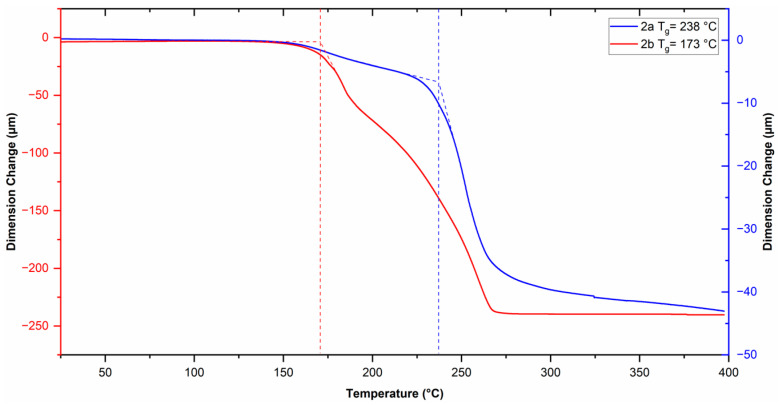
*TMA* thermograms of the sulfonyl-containing polynorbornene dicarboximides (Dashed lines indicate *T_g_* values).

**Figure 6:**
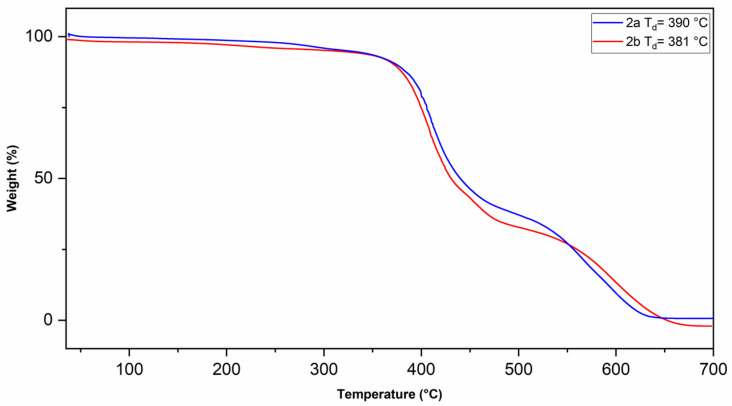
*TGA* thermograms of the sulfonyl-containing polynorbornene dicarboximides.

The physical properties of the polymers are summarized in Table 1. The glass transition temperature (*T_g_*) of the polymers was measured by TMA (Figure 5). A *T_g_* value of 238 °C was observed for polymer **2a** and 173 °C for polymer **2b**. The decrease in the *T_g_* value for polymer **2b** compared to that of **2a** is ascribed to the presence of the oxygen heteroatom in the cyclopentane ring of the polymer backbone, which increases the conformational mobility of the polymer main chain, thus achieving the relaxation process at a lower temperature. The onset of decomposition temperature (*T_d_*) of the polymers was measured by TGA (Figure 6). The thermal profiles shifted 2% relative to each other, starting from the one corresponding to polymer **2a**, to clearly follow the thermal decomposition curves of both polymers. A *T_d_* value of 390 °C was observed for polymer **2a** and 381 °C for polymer **2b**, this indicates that these polymers possess relatively high thermal stability. The small difference in the thermal stability of the polymers is attributed to the ether-like bond in polymer **2b**, which has a lower bond energy compared to that of the C–H bond [46].

The elastic modulus (*E*) and the tensile strength (*σ*) of the polymers were measured by stress–strain tests in tension on the prepared films. According to Table 1, polymer **2a** has a higher elastic modulus and tensile strength (*E* = 2123 MPa and *σ* = 63.5 MPa) compared to polymer **2b** (*E* = 2078 MPa and *σ* = 59.3 MPa). This observation indicates that the presence of an oxygen heteroatom in the polymer backbone facilitates chain relaxation by enabling the polymer to adopt different conformations. This is reflected in lower thermomechanical properties compared to the homologous polymer that lacks oxygen heteroatoms.

The density, *ρ*, of the polymers was measured in film form using the flotation method at room temperature in an ethanol medium. The density values reported in Table 1 indicate that the presence of an oxygen heteroatom in the cyclopentane ring of polymer **2b** enhances chain packing efficiency, as reflected by its higher density and lower fractional free volume (*FFV*) compared to polymer **2a**. In contrast, polymer **2a** appears to have more restricted backbone mobility, as a result of the presence of the methylene group in the cyclopentane ring of the polymer’s main chain; therefore, this polymer displays the lowest density and the highest *FFV* among the two polymers examined in this study.

The *FFV* was determined using the Bondi group contribution method [14] according to the following equation:

*FFV* = (*V* − *V*_0_)/*V* (4)

where *V* denotes the specific volume (1/*ρ*), and *V*_0_ represents the specific occupied volume, which can be calculated from the Van der Waals volume *V_w_* as *V*_0_ = 1.3 *V_w_*, estimated using van Krevelen’s data [47].

**Figure 7:**
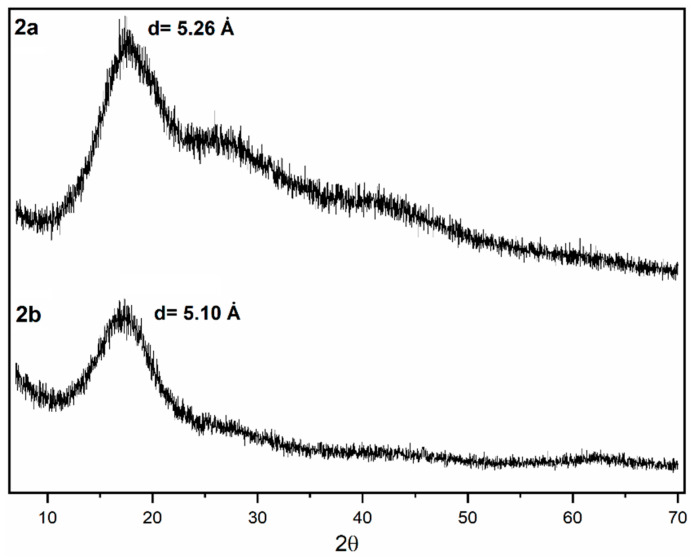
*XRD* patterns of the sulfonyl-containing polynorbornene dicarboximides.

X-ray diffraction (XRD) measurements (Figure 7) performed on sulfonyl-containing polymer films display patterns typical of dense amorphous polymers such as polynorbornene dicarboximides, in which a broad diffraction trace can be clearly identified showing a maximum around 17° (2*θ*). Two additional diffraction halos can also be observed at approximately 28° and 42° (2*θ*) and may be attributed to the coexistence of different levels or types of structural order within the material. Each halo corresponds to a distinct characteristic spacing (*d-spacing*): a low-angle halo is commonly associated with longer-range correlations related to interchain packing, whereas a high-angle halo is associated with short-range distances arising from local segmental interactions. On the one hand, the structural heterogeneity of this class of polymers is likely to result in the appearance of more than one diffraction halo, since polynorbornene dicarboximides contain both an aliphatic, hydrophobic moiety formed by the cyclopentane ring and an aromatic, hydrophilic moiety located in the imide region. Furthermore, the membrane casting process may induce partial orientation of the polymer chains, leading to the separation of halos associated with specific packing directions. Likewise, additional halos may arise from specific correlations, such as the π−π interactions discussed above, which are associated with the charge transfer (CT) interaction formed by π electrons of ring arrangements in polynorbornene dicarboximides. From the XRD patterns, the mean intersegmental distance or chain packing density, *d-spacing*, of polymers **2a** and **2b** can be estimated. To accomplish this, the Bragg’s equation is used, n*λ* = 2*d*sin*θ* [48], taking the angle at which the reflective intensity in the amorphous peak is maximum. As shown in Table 1, the average *d-spacing* and *FFV* values are well correlated. In other words, a larger *d-spacing* is expected to result in a higher *FFV*, and vice versa.

**Figure 8:**
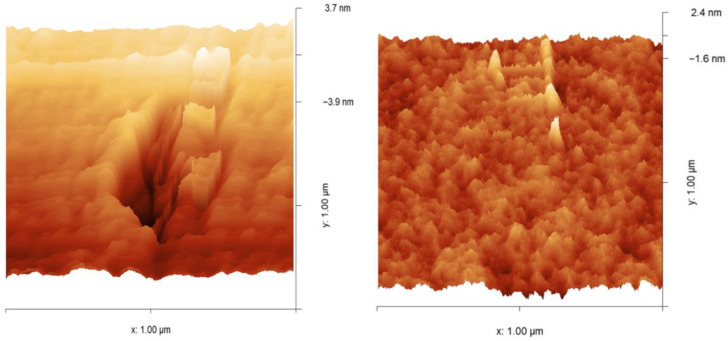
Three-dimensional *AFM* micrographs (1 × 1 μm) of polymer **2a** (**left**) and polymer **2b** (**right**).

In Figure 8, the surface morphologies of polymer **2a** (left) and polymer **2b** (right), obtained by the Grubbs 2nd generation catalyst (**I**), are observed by tapping mode atomic force microscopy in three dimensions. Polymer **2a** exhibits an irregular surface morphology characterized by large cavities per unit area, which may be attributed to reduced main-chain mobility arising from steric hindrance of the –CH_2_– group in the cyclopentane ring of the ROMP-prepared polymer. The latter leads to lower efficiency in the packing of polymer chains, which in turn is reflected in lower density. Polymer **2b** shows a disrupted surface morphology that lacks cavities, suggesting that the presence of an oxygen heteroatom in the cyclopentane ring of polymer **2b** increases chain mobility, leading to enhanced chain packing ability, which is reflected in a higher density. The absence of cavities also accounts for the lower *FFV* of this polymer compared to polymer **2a**.

Table 2 presents the permeability (*P*), diffusion (*D*), and solubility (*S*) values of different gases measured at 30 °C and an upstream pressure of 1 atm for membranes of sulfonyl-containing polynorbornene dicarboximides. In general, the permeability coefficients of sulfonyl-containing polynorbornene dicarboximides are observed to follow the order *P*(H_2_) > *P*(CO_2_) > *P*(O_2_) > *P*(C_2_H_4_) > *P*(N_2_) ≥ *P*(CH_4_) > *P*(C_3_H_6_). This trend differs from that of the diffusion coefficients, which decrease in the sequence *D*(H_2_) > *D*(O_2_) > *D*(N_2_) ≥ *D*(CO_2_) > *D*(CH_4_) > *D*(C_2_H_4_) ≥ *D*(C_3_H_6_). Table 2 clearly shows that the presence of the sulfonyl group decreases the gas permeability coefficient of both polymeric membranes studied here as a consequence of the decrease mainly in gas diffusion compared to that of membrane **3c**, which corresponds to a polynorbornene dicarboximide that lacks sulfonyl group. The latter suggests that the strong electron-withdrawal exerted by the sulfonyl group leads to greater efficiency in the packing of polymer chains, which in turn is reflected in lower permeabilities, as reveals the fact that the gas permeability coefficients in the sulfonyl-containing **2a** membrane have values ranging from 27% (H_2_) to 65% (C_3_H_6_) lower than those of membrane **3c**, which lacks sulfonyl group. In addition, it is also observed that the presence of an oxygen heteroatom in the cyclopentane ring of the sulfonyl-containing **2b** membrane further decreases gas permeability, showing values between 48% (H_2_) and 86% (C_3_H_6_) lower than those of membrane **3c**. On the other hand, when comparing the gas permeability coefficients of membranes **2a** and **2b**, which contain sulfonyl groups, it is observed that membrane **2b** is less permeable. This decrease in gas permeability of membrane **2b** is due to both the diffusion process and the gas sorption stage. The lowering in diffusion coefficients is mainly due to a lower *FFV* associated with the greater chain packing efficiency of polymer **2b** compared to polymer **2a**, which in turn hinders the diffusion of gas molecules through the polymer. In general, as shown in Table 2, the apparent solubility coefficients for membrane **2b** are lower than those found for membrane **2a**. It is well known that non-equilibrium excess free volumes are a key feature of glassy polymers, thus, the lower solubility of the gas can be directly attributed to the smaller amount of this additional free volume in which sorption processes can take place. In this regard, Table 1 shows that polymer **2b** has a lower *FFV* than polymer **2a**, which correlates quite well with the previous statement. Commonly, the dual-mode model has been employed to interpret gas sorption in glassy polymers. This approach assumes that the glassy state consists of a continuous phase with dispersed microcavities, which explains the excess volume. Solubility in the continuous phase follows Henry’s law, whereas microcavities function as Langmuir sites where processes such as adsorption occur. The sorption at Langmuir sites is often employed to correlate the amount of non-equilibrium excess free volume in the glassy state [49]. According to the dual-mode model, Langmuir sorption in glassy polymer membranes depends on the difference between *T_g_* and *T*; therefore, as the *T_g_* of the polymer decreases, the nonequilibrium excess free volume also decreases at a given temperature *T* [50]. Based on the above, the relatively low *S* values found for membrane **2b** used in this research could also be due to the proximity of the glass transition temperature of the sulfonyl-containing polymer to the working temperature. As can be seen from Table 2, carbon dioxide and the hydrocarbon gases of greater condensability exhibit the largest apparent solubility coefficients, *S*, as determined from the *P*/*D* ratio, so that for polymer **2a** the following trends are observed: *S*(C_3_H_6_) > *S*(CO_2_) > *S*(C_2_H_4_) > *S*(CH_4_) > *S*(O_2_) > *S*(N_2_) > *S*(H_2_), while for polymer **2b** the trends are: *S*(CO_2_) > *S*(C_2_H_4_) > *S*(CH_4_) > *S*(C_3_H_6_) > *S*(O_2_) ≥ *S*(N_2_) > *S*(H_2_). These solubility trends suggest that the bulky methylene group in **2a** is much more effective for the sorption of C_3_H_6_ compared to the oxygen heteroatom in **2b**. This fact is related to the strong affinity of olefins for less polar polymer matrices [51]. The large difference in the solubility coefficient of propylene between the two polymers is due both to the greater sorption of this gas in polymer **2a**, attributed to polymer-penetrant interactions, and to the greater diffusion of C_3_H_6_ in polymer **2b**. Further research, including the calculation of solubility activation energy (*E_S_*) and diffusion activation energy (*E_D_*), is needed to clarify this issue.

The effects of the sulfonyl group and the oxygen heteroatom on the performance of polynorbornene dicarboximide membranes were also evaluated using the permselectivity coefficient, or ideal separation factor (*α*) for gas A over gas B, which serves as an indicator of a polymer membrane’s ability to separate a specific gas mixture and is defined as:

(5) $$\alpha \left(A/B\right)=\;\frac{P(A)}{P(B)}=\;\frac{D(A)}{D(B)}\times \;\frac{S(A)}{S(B)}$$

Equation (5), defined as the ratio of the pure-gas permeability coefficients *P*(A)/*P*(B), points out that the discriminative effects governing gas transport in membranes may arise from the diffusion stage, the solubility step, or a combination of both. Accordingly, the ideal separation factor can be decomposed into two contributions: a diffusivity selectivity term, *α*_D_ = *D*(A)/*D*(B), and a solubility selectivity term, *α*_S_ = *S*(A)/*S*(B). This approach enables determination of whether *α*_D_ or *α*_S_ plays the dominant role in achieving separation of a given gas pair.

Table 3 compares the permselectivities of different gas pairs for membranes **2a** and **2b**. As shown, both sulfonyl-containing polynorbornene dicarboximide membranes, which exhibit lower gas permeability coefficients, display higher ideal separation factors than membrane **3c**, which lacks the sulfonyl group. This behavior reflects the trade-off commonly observed in glassy polymers, whereby lower permeability is accompanied by higher permselectivity. For instance, the permselectivity coefficients *α*(CO_2_/CH_4_) for membranes **2a** and **2b** increase by approximately 35% and 23%, respectively, at the expense of the CO_2_ permeability coefficient, which decreases by approximately 27% and 51%, respectively.

Interestingly, when the two sulfonyl-containing membranes are compared to each other, it is seen that membrane **2a** not only increases the permeability coefficient of O_2_ by 54%, of N_2_ by 42%, of CO_2_ by 51% and of CH_4_ by 37%, but also augments the permselectivity coefficient *α*(O_2_/N_2_) by almost 7% and *α*(CO_2_/CH_4_) by almost 10%, among others. In addition to exhibiting separation performances for O_2_/N_2_, CO_2_/CH_4_, and CO_2_/N_2_ comparable to those of membrane **2a**, membrane **2b** also displays the highest permselectivity coefficients for the separation of H_2_ from low-molecular-weight hydrocarbon gases such as C_2_H_4_ and C_3_H_6_. In this regard, *α*(H_2_/C_3_H_6_) is almost double that of membrane **2a** and is mainly due to an increase in the solubility selectivity contribution, *α*_S_, which in the case of this pair of gases is approximately eight times greater than in membrane **2a**.

In this context, it is worth noting that *α*(H_2_/C_3_H_6_) = 112.4 for membrane **2b** represents the highest separation factor reported to date for polynorbornene dicarboximides and the value that most closely approaches this result is only obtained by the CF_3_-*meta* substitution of the phenyl group in this type of polymers [52]. Furthermore, it was found that *α*_D_ = *D*(H_2_)/*D*(C_3_H_6_) = 8034 for membrane **2a**, since *α*_D_ ˃˃˃ *α*_S_ the diffusivity selectivity makes the greatest contribution to achieving the separation of the gas pair. However, the relatively low solubility of hydrogen compared to that of propylene is responsible for the membrane’s moderate discriminatory performance in H_2_/C_3_H_6_ separation. As mentioned above, the contribution of solubility to permselectivity is expressed as *α*_S_ = *S*(A)/*S*(B), where A and B denote the gases to be separated. Examination of the data in Table 3 indicates that solubility is a poor contributor to permselectivity for condensable gases. For example, when propylene is taken as gas A in the most permeable membrane (**2a**), the *α*_S_ values are lower than 2 when gas B is ethylene or carbon dioxide. In contrast, the solubility selectivity shows good performance for less condensable gases, with *α*_S_ values reaching up to 33 and 24 for nitrogen and oxygen, respectively.

## 4. Conclusions

The synthesis and subsequent ROMP of two new homologous sulfonyl-containing polynorbornene dicarboximide monomers was successfully carried out, yielding materials with high thermal and mechanical properties. The properties of polymers are influenced by the presence of a methylene group or an oxygen heteroatom in the cyclopentane ring of the polymer backbone, primarily due to steric hindrance affecting the main chain’s mobility. The presence of the methylene group increases the thermomechanical properties of the polymer, while its substitution by an oxygen heteroatom has the opposite effect. The gas transport properties of the sulfonyl-containing polynorbornene dicarboximides **2a** (with methylene group) and **2b** (with oxygen heteroatom) were compared with those of a polynorbornene dicarboximide lacking sulfonyl groups. It is observed that the presence of sulfonyl groups in both polymers decreases the gas permeability of their corresponding membranes as a consequence of the decrease mainly in gas diffusion. Moreover, the presence of the oxygen heteroatom in polymer **2b** further decreases gas permeability compared to polymer **2a**, generally as a consequence of the decrease in both gas diffusion and gas solubility, attributed mainly to a lower *FFV*. It was also found that the polymer **2b** membrane has one of the highest permselectivity coefficients reported to date for the separation of H_2_/C_3_H_6_ in glassy polynorbornene dicarboximides.

## Figures and Tables

**Table 1:** Physical properties of sulfonyl-containing polynorbornene dicarboximides.

Polymer	*T_g_* [°C]	*T_d_* [°C]	*σ* [MPa]	*E* [MPa]	*ρ* [g cm^−3^]	*FFV*	*d-spacing* [Å]
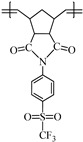 **2a**	238	390	63.5	2123	1.405	0.178	5.26
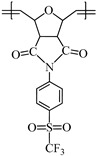 **2b**	173	381	59.3	2078	1.490	0.152	5.10

**2a**: poly(*N*-4-(trifluoromethylsulfonyl)phenyl-norbornene-5,6-dicarboximide); **2b**: poly(*N*-4-(trifluoromethylsulfonyl)phenyl-7-oxanorbornene-5,6-dicarboximide); *T_g_*: glass transition temperature; *T_d_*: onset of decomposition temperature; *σ*: tensile strength; *E*: Young’s modulus; *ρ*: density; *FFV*: fractional free volume; *d-spacing*: mean intersegmental distance.

**Table 2:** Permeability (*P*), diffusion (*D*), and solubility (*S*) coefficient values of several gases measured at 30 °C and an upstream pressure of 1 atm for membranes of sulfonyl-containing polynorbornene dicarboximides.

Polymer	Gas	*P* [Barrers]	*D* × 10^8^ [cm^2^ s^−1^]	*S* × 10^3^ [cm^3^ cm^−3^ cmHg^−1^]
** 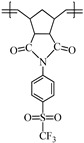 ** **2a**	H_2_	25.02	167.12	1.49
	N_2_	0.87	1.48	5.90
	O_2_	3.94	4.76	8.29
	CO_2_	25.05	1.36	183.66
	CH_4_	0.85	0.33	25.44
	C_2_H_4_	1.37	0.11	129.38
	C_3_H_6_	0.41	0.020	199.62
** 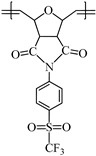 ** **2b**	H_2_	17.66	140.88	1.25
	N_2_	0.61	0.89	6.82
	O_2_	2.56	3.72	6.88
	CO_2_	16.58	0.96	172.72
	CH_4_	0.62	0.23	27.15
	C_2_H_4_	0.78	0.077	99.94
	C_3_H_6_	0.16	0.083	18.79
** 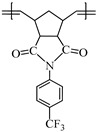 ** **3c**	H_2_	34.42	252.29	1.4
	N_2_	1.47	2.78	5.3
	O_2_	6.17	8.55	7.2
	CO_2_	34.37	2.16	159.5
	CH_4_	1.59	0.75	21.0
	C_2_H_4_	2.40	0.18	133.0
	C_3_H_6_	1.20	0.041	286.9

*P*: permeability coefficient; *D*: diffusion coefficient; *S*: apparent solubility coefficient; H_2_: hydrogen; N_2_: nitrogen; O_2_: oxygen; CO_2_: carbon dioxide; CH_4_: methane; C_2_H_4_: ethylene; C_3_H_6_: propylene; **2a**: poly(*N*-4-(trifluoromethylsulfonyl)phenyl-norbornene-5,6-dicarboximide); **2b**: poly(*N*-4-(trifluoromethylsulfonyl)phenyl-7-oxanorbornene-5,6-dicarboximide); **3c**: poly(*N*-4-trifluoromethylphenyl-*exo*-*endo*-norbornen-5,6-dicarboximide) [52].

**Table 3:** Ideal gas separation factors (*α*) determined at 30 °C for various gas pairs in membranes **2a** and **2b**.

Polymer	*α*_O2/N2_	*α*_CO2/CH4_	*α*_CO2/N2_	*α*_C2H4/C3H6_	*α*_H2/CH4_	*α*_H2/C2H4_	*α*_H2/C3H6_
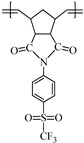 **2a**	4.5	29.3	28.6	3.3	29.3	18.3	60.3
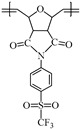 **2b**	4.2	26.7	27.3	4.8	28.5	22.7	112.4
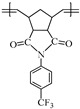 **3c**	4.2	21.6	23.3	2.0	21.6	14.3	28.7

H_2_: hydrogen; N_2_: nitrogen; O_2_: oxygen; CO_2_: carbon dioxide; CH_4_: methane; C_2_H_4_: ethylene; C_3_H_6_: propylene; **2a**: poly(*N*-4-(trifluoromethylsulfonyl)phenyl-norbornene-5,6-dicarboximide); **2b**: poly(*N*-4-(trifluoromethylsulfonyl)phenyl-7-oxanorbornene-5,6-dicarboximide); **3c**: poly(*N*-4-trifluoromethylphenyl-*exo*-*endo*-norbornen-5,6-dicarboximide) [52].

## Data Availability

The original contributions presented in this study are included in the article. Further inquiries can be directed to the corresponding author.

## Abbreviations

The following abbreviations are used in this manuscript:

**1a**	*N*-4-(trifluoromethylsulfonyl)phenyl-norbornene-5,6-dicarboximide
**1b**	*N*-4-(trifluoromethylsulfonyl)phenyl-7-oxanorbornene-5,6-dicarboximide
**2a**	poly(*N*-4-(trifluoromethylsulfonyl)phenyl-norbornene-5,6-dicarboximide)
**2b**	poly(*N*-4-(trifluoromethylsulfonyl)phenyl-7-oxanorbornene-5,6-dicarboximide)
**I**	Grubbs 2nd generation catalyst
NMR	Nuclear magnetic resonance
FT-IR	Fourier transform infrared spectroscopy
TMA	Thermomechanical analysis
TGA	Thermogravimetric analysis
AFM	Atomic force microscopy
XRD	X-ray diffraction
ROMP	Ring-opening metathesis polymerization
*T_g_*	Glass transition temperature
*T_d_*	Onset of decomposition temperature
*E*	Young’s modulus
*σ*	Tensile strength
*ρ*	Density
NDA	Norbornene-5,6-dicarboxylic anhydride
*P*	Permeability coefficient
*D*	Diffusion coefficient
*S*	Apparent solubility coefficient
*FFV*	Fractional free volume
*d-spacing*	Mean intersegmental distance
*α*	Permselectivity coefficient
